# Discovery of the photosynthetic relatives of the "Maltese mushroom" *Cynomorium*

**DOI:** 10.1186/1471-2148-5-38

**Published:** 2005-06-21

**Authors:** Daniel L Nickrent, Joshua P Der, Frank E Anderson

**Affiliations:** 1 Department of Plant Biology, Southern Illinois University, Carbondale, IL 62901-6509, USA; 2 Department of Zoology, Southern Illinois University, Carbondale, IL 62901-6501, USA

## Abstract

**Background:**

Although recent molecular phylogenetic studies have identified the photosynthetic relatives of several enigmatic holoparasitic angiosperms, uncertainty remains for the last parasitic plant order, Balanophorales, often considered to include two families, Balanophoraceae and Cynomoriaceae. The nonphotosynthetic (holoparasitic) flowering plant *Cynomorium coccineum *has long been known to the Muslim world as "tarthuth" and to Europeans as the "Maltese mushroom"; *C. songaricum *is known in Chinese medicine as "suo yang." Interest in these plants is increasing and they are being extensively collected from wild populations for use in herbal medicines.

**Results:**

Here we report molecular phylogenetic analyses of nuclear ribosomal DNA and mitochondrial *matR *sequence data that strongly support the independent origin of Balanophoraceae and Cynomoriaceae. Analyses of single gene and combined gene data sets place *Cynomorium *in Saxifragales, possibly near Crassulaceae (stonecrop family). Balanophoraceae appear related to Santalales (sandalwood order), a position previously suggested from morphological characters that are often assumed to be convergent.

**Conclusion:**

Our work shows that *Cynomorium *and Balanophoraceae are not closely related as indicated in all past and present classifications. Thus, morphological features, such as inflorescences bearing numerous highly reduced flowers, are convergent and were attained independently by these two holoparasite lineages. Given the widespread harvest of wild *Cynomorium *species for herbal medicines, we here raise conservation concerns and suggest that further molecular phylogenetic work is needed to identify its photosynthetic relatives. These relatives, which will be easier to cultivate, should then be examined for phytochemical activity purported to be present in the more sensitive *Cynomorium*.

## Background

Molecular phylogenetics has expanded understanding of relationships among all major angiosperm groups and has thereby strongly impacted their classification [1]. More recently, such advances have also included some nonphotosynthetic holoparasites whose phylogenetic positions had previously been uncertain, such as Hydnoraceae [2] and Rafflesiales [3]. The latter study documented that Rafflesiales are actually a polyphyletic assemblage of three or four independent evolutionary lineages. The losses and reductions in features that are pervasive in parasitic plants have resulted in remarkable morphological convergences, thereby explaining their erroneous placement by traditional methods. Previous molecular phylogenetic work with such holoparasites highlighted the need to employ gene sequences from different subcellular compartments and analytical methods that accommodate rate heterogeneity, thus avoiding long-branch attraction artifacts [3]. These steps are justified because congruence among different gene trees provides evidence that the organismal tree is being recovered, while incongruence suggests the presence of nonstandard processes such as introgression, lineage sorting, and horizontal gene transfer (HGT) [3-6].

**Figure 1:**
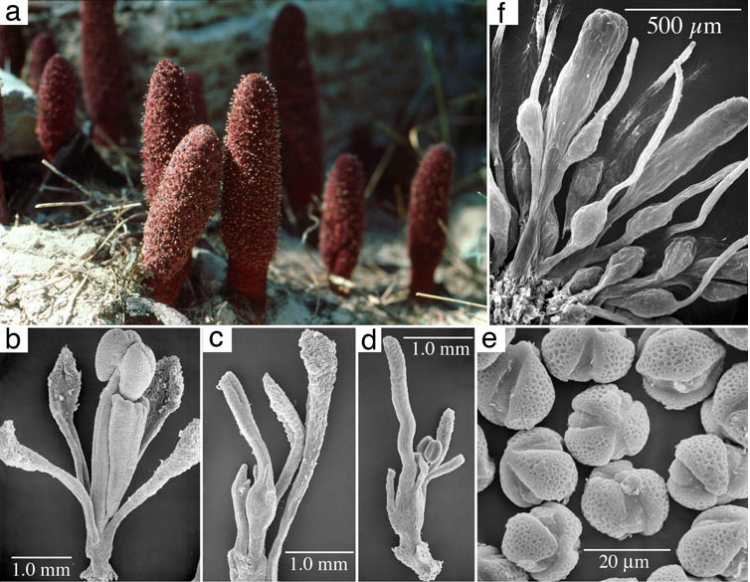
**1 Morphology of *Cynomorium coccineum *(a-e) and *Balanophora fungosa *(f)**. a, Habit showing inflorescences emerging from the ground. b, Scanning electron micrograph (SEM) of staminate flower. c, SEM of carpellate flower. d, SEM of bisexual flower. e, SEM of pollen. f, SEM of carpellate flowers of *Balanophora *showing remarkable convergence with those of *Cynomorium*.

In addition to the abovementioned holoparasites, another group with uncertain placement is Balanophorales. For over 150 years, there has been taxonomic debate as to whether the genus *Cynomorium *is part of Balanophoraceae [7,8] or whether it should be classified as a separate family, Cynomoriaceae [9]. These plants are fleshy, monoecious or dioecious holoparasitic herbs that often produce swollen tuberous haustorial root connections to their host plants. Their stems bear scalelike leaves and their unisexual flowers represent the ultimate in reduction among angiosperms [10] (Figure 1). Balanophoraceae (in the strict sense) contains 17 genera and 44 species in the neo- and paleotropics whereas *Cynomorium *has two species in eastern Asia (*C. songaricum*) and the Mediterranean (*C. coccineum*). The presence of bisexual flowers, an unusual bimodal karyotype [11] and features of the stamens, ovules, and embryo sac [12] in *Cynomorium *all support its segregation from Balanophoraceae. Whether considered one or two families, all past and present classifications accept a relationships between *Cynomorium *and Balanophoraceae. Previous molecular phylogenetic work using only nuclear small-subunit ribosomal DNA [13] suggested placement of *Cynomorium *with Saxifragales and Balanophoraceae with the sandalwood order (Santalales). The validity of these results could not be assessed because high substitution rates in these plants could potentially confound phylogeny estimation [14] and because confirmatory data from other genes was lacking. Here we utilize both nuclear and mitochondrial gene sequence data, analyzed with maximum parsimony (MP) and Bayesian inference (BI) methods, to determine whether Balanophoraceae and Cynomoriaceae are related to one another and where these clades fit in the global angiosperm phylogeny.

## Results

**Figure 2:**
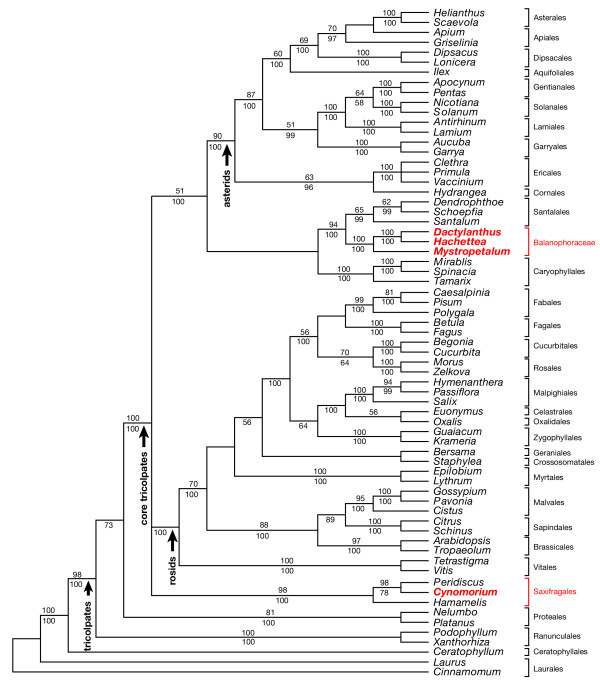
**Maximum parsimony strict consensus tree from the *global *data set**. Data set of combined nuclear SSU rDNA, chloroplast *rbcL *and *atpB *and mitochondrial *matR*. Strict consensus of 4 trees. Bootstrap support values are shown above the lines, Bayesian posterior probabilities below. The minimum length tree was 9405 steps (consistency index minus uninformative sites 0.3126, retention index 0.4301, rescaled consistency index 0.1709). *Cynomorium *is not part of Balanophoraceae but a taxon within Saxifragales.

**Figure 3:**
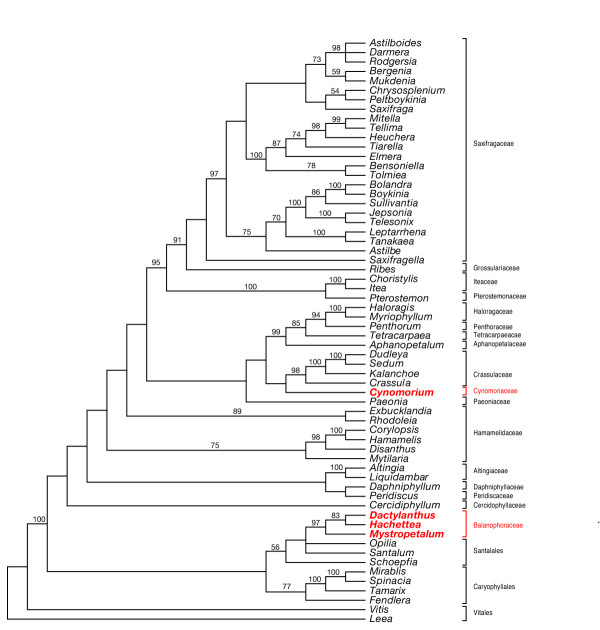
**Maximum parsimony strict consensus tree from the *Saxifragales *data set**. Data set of combined nuclear SSU and LSU rDNA, chloroplast *rbcL*, *atpB*, and *matK*. Strict consensus of 9 trees. Bootstrap support values are shown above the lines. The minimum length tree was 8282 steps (consistency index minusuninformative sites 0.3975, retention index 0.5609, rescaled consistency index 0.2750). Although the sister relationship between *Cynomorium *and Crassulaceae is seen in the shortest trees, bootstrap support for this clade is low.

Results of analyses of of the global data set using both MP (Figure 2) and BI (Additional File 3) strongly indicate that *Cynomorium *is not closely related to Balanophoraceae but is a component of Saxifragales. Small-subunit (SSU) nuclear ribosomal DNA alone analyzed with BI (Additional File 4) and ML (Additional File 5) gave the same result as obtained with the global data set. Similarly, mitochondrial *matR *analyzed alone with BI (Additional File 6) showed *Cynomorium *well separated from Balanophoraceae. Moreover, strong support is obtained from multigene and separate gene analyses for a relationship between Balanophoraceae and Santalales. Given these results, attempts were made to precisely determine the sister taxon of *Cynomorium *within Saxifragales, a clade that has been subjected to extensive molecular phylogenetic work [15,16]. The shortest MP trees contained a clade composed of *Cynomorium *and Crassulaceae (Figure 3), however, this relationship did not receive high bootstrap (BS) support. A similar lack of resolution for this clade was also seen in the BI tree (Additional File 7). On both the MP and BI trees, *Cynomorium *is part of a clade with moderate support that contains Crassulaceae, Paeoniaceae, Aphanopetalaceae, Tetracarpaeaceae, Haloragaceae, Pterostemonaceae, Iteaceae, Grossulariaceae, and Saxifragaceae.

## Discussion

This work documents that *Cynomorium *and Balanophoraceae are not closely related, a result in conflict with past and present classifications. Assuming our molecular gene trees reflect the organismal tree, these data indicate that morphological features, such as inflorescences bearing numerous highly reduced flowers, are convergent and were thus attained independently by these two holoparasite lineages.

### *Cynomorium *in Saxifragales

From an historical perspective, Hooker [7] allied *Cynomorium *and Balanophoraceae with Haloragaceae (the latter is a family in Saxifragales, see Figure 3) because both groups have epigynous flowers with stamens opposite the valvate perianth lobes. He also noted the striking similarities between the female flowers of *Gunnera *and *Lophophytum *(Balanophoraceae). Our analyses that included *Gunnera *(data not shown) indicate it is not closely related to either Cynomoriaceae or Balanophoraceae, a result in agreement with other work that placed *Gunnera *as sister to all core eudicots [17,18]. Other 19^th ^century workers such as Hoffmeister proposed a relationships between *Cynomorium *and *Hippuris *(Plantaginaceae, an asterid) because both have a single, epigynous stamen attached to the top of a unicarpellate ovary. This relationship was not confirmed following a detailed floral morphological study [19] nor was *Cynomorium *shown to be related to asterids following molecular analyses reported here.

The exact position of Saxifragales within the core eudicot clade remains uncertain because various molecular phylogenetic analyses are in conflict (c. f. [17,18,20,21]). These studies have proposed relationships with rosids, asterids, caryophyllids, and Santalales, i.e. nearly all members of the core tricolpate clade (see review by Judd and Olmstead [22]). The results reported here also demonstrate uncertainty with regard to the topology of the above clades (Figure 2). Given the variety of ways these clades could resolve, the possibility exists that Balanophoraceae and *Cynomorium *are more closely related than graphically depicted in Figure 2. Although future molecular phylogenetic studies may support this, it is unlikely that these two holoparasite groups will become sister given that the Balanophoraceae + Santalales clade has BS support of 94% and *Cynomorium *is sister to *Peridiscus *with 98% BS support. Moreover, the latter two genera are sister to *Hamamelis *with a 98% BS value.

Long-branch attraction has long been suggested as a confounding factor when conducting phylogenetic analyses of organisms with marked among-lineage rate heterogeneity [23]. Indeed, our group has shown that Rafflesiales, another holoparasitic angiosperm group, is susceptible to long-branch attraction, particularly when analyzed with MP [3]. The results reported here differ from that study in several respects. First, MP analysis of the global data set does not indicate that Balanophoraceae and *Cynomorium *exhibit particularly long branches with respect to other angiosperms, nor are these holoparasite clades "attracted" to each other (Additional File 8). This result differs from that obtained when the Rafflesiales molecular data are analyzed with MP where the four distinct lineages (Apodanthaceae, Cytinaceae, Mitrastemonaceae and Rafflesiaceae) appear monophyletic [3]. When the global data set is analyzed with BI, some degree of branch length heterogeneity is seen (Additional File 9), however, Balanophoraceae and *Cynomorium *still remain on distinct clades. It should be pointed out that for the global data set, branch length comparisons between holoparasites and photosynthetic plants are somewhat biased given that the chloroplast genes were coded as missing for the holoparasites. Taken together, we feel our results are not being influence by long-branch attraction and that they provide strong evidence for the independent evolution of these two holoparasite lineages.

Attempts to find morphological features that link *Cynomorium *with Saxifragales are complicated, first because the latter order is morphologically heterogeneous and second because of morphological reductions in the holoparasite (see below). The heterogeneity of Saxifragales is evidenced by the fact that pre-molecular classifications placed many of its component families in distinct subclasses [8]. Although a close relationship between *Cynomorium *and Crassulaceae was not seen on the BI tree, the shortest MP tree showed these genera as sister taxa, thus this possible affinity will here be considered. Tricolporate pollen (Figure 1e), stem succulence, and proanthocyanidins (red pigments, see [24]) are possible characters that may reflect a relationship between *Cynomorium *and Crassulaceae, but these are all general features found in many groups. Other than such facies, there are few floral characters that could be considered synapomorphies between the extremely reduced flowers of *Cynomorium *and those of Crassulaceae. The staminate flower of *Cynomorium *is composed of four to six distinct perianth parts forming a whorl below the single stamen and a wedge-shaped structure interpreted as a pistillode [25]. The apical portion of the pistillode is concave and accepts the base of an anther theca prior to filament elongation (Figure 1b) and its internal lateral surface has a groove that accepts the filament. Stamen morphology is not unlike many tricolpate angiosperms: the anthers are dorsifixed, tetrasporangiate, dithecal and open by longitudinal slits. The carpellate flower (Figure 1c) is composed of a single carpel with an elongated, grooved style. The perianth (or perigonial scales) are much smaller than in the staminate flower, reduced to small papillae at the summit of the ovary or along its sides. The bisexual flowers are similar to carpellate flowers except for the addition of a stamen at the base of the ovary (Figure 1d). As with many holoparasitic flowering plants, the reduction or loss of morphological features interferes with the comparative morphological approach when searching for relatives among less modified photosynthetic plants.

### Balanophoraceae in Santalales

Although precedents exists for placing Balanophoraceae with Santalales [8,26-29], recovering this relationship with both nuclear and mitochondrial genes is surprising because any morphological similarities (e.g., reduction in the gynoecium) have usually been assumed to be cases of convergence [30]. Moreover, BI of the Saxifragales data set indicates a derivation of Balanophoraceae from *within *Santalales, not as its sister (Figure 3 and Additional File 8). Although a multigene analysis of Santalales with robust taxon sampling exists [31], it was generated with nuclear ribosomal DNA and chloroplast genes. To conduct analyses with Balanophoraceae and Santalales, mitochondrial genes from the latter will be needed to test relationships.

Cronquist [8] viewed the sandalwoods as the best candidate ancestral group for Balanophoraceae, however, he admitted any similarities might reflect convergent adaptations to parasitism. Of the 17 genera in the family, only *Dactylanthus, Hachettea *and *Mystropetalon *were used in this study because they are sister (and basal) to the remaining genera and have lower substitution rates in SSU rDNA, thus avoiding potential long-branch artifacts. The floral morphology of *Mystropetalon *is less reduced than other genera in the family. Given that its staminate flowers have pistillodes (vestigial carpels) and carpellate flowers have staminodes (vestigial stamens), it is assumed this plesiomorphic condition reflects evolution from an ancestor with bisexual flowers. Floral features in common with Santalales include a valvate perianth and stamens with "typical" morphology (i.e. anther sacs and filaments as opposed to synangia as seen in *Balanophora*). When examining the haustorial structure of *Mystropetalon*, Weber [32] noted the presence of a collapsed zone, graniferous tracheary elements, and "runners," which he associated with Santalales. Similarly, reduction in ovules is seen in several santalalean families, particularly the mistletoes in Viscaceae where no true ovule exists. Either an embryo sac is embedded within central placenta called a "mamelon" or the archesporium develops hypodermally in the ovary, functioning directly as megaspore mother cells [33]. Similarly, ovules have been lost in some Santalaceae (*Exocarpos*) and Loranthaceae. During the course of floral reduction, the loss of ovules most influenced Fagerlind [27] who drew a direct ancestor-descendant relationship between Santalaceae and Balanophoraceae.

As mentioned above, such apparent similarities between Balanophoraceae and Santalales have also been interpreted as convergent. Kuijt [10] suggests that the reduction of santalalean ovules may permit retention of meristematic activity, a requirement for the formation of complex fruits in epiparasites. For root parasites such as Balanophoraceae, he suggests ovular reduction is related to selection for tiny seeds that require host stimulants for germination, as is seen in Orobanchaceae. In addition to ovules, the androecia of both Viscaceae and Balanophoraceae are diverse with both showing trends toward the formation of synandria and porose dehiscence. Whether these trends are convergent or follow from homologous suites of genes may yield to direct testing using floral homeotic mutants.

Although herbaceous root parasites have evolved in Santalaceae (e.g. *Thesium, Quinchamalium*), most members of Santalales are woody. Completely holoparasitic species do not occur in this order, with the possible exception of *Daenikera*, an endemic monotypic genus of New Caledonia whose photosynthetic capabilities remain to be determined. As adults, dwarf mistletoes (*Arceuthobium*, Viscaceae) fix only about 30% of the carbon needed for growth [34,35] but prior to host attachment seedlings actively photosynthesize. For this reason these mistletoes must be considered advanced obligate hemiparasites, not holoparasites. Thus, to include with Santalales the entirely holoparasitic group Balanophoraceae requires further investigation to identify the exact sister group.

### Ethnobotany and conservation biology of *Cynomorium*

*Cynomorium coccineum*, known to the Muslim world as "tarthuth," has been harvested from the deserts of north Africa and the Middle East for thousands of years. Arabs and Bedouins eat the interior portions of fresh young stems, prepare infusions of older stems to treat colic or stomach ulcers, or dry and pulverize the plant for use as a spice or condiment with meat dishes [36]. Medicinal uses of tarthuth can be traced to Al-Kindi, Al-Razi (Rhazes), Ibn Masawayh, Ibn Wahshiya, and Maimonides but the plant became known to Europeans only in the 16^th ^century. A group called the Knights Hospitaller of St. John operated a hospital in Jerusalem and learned of the medicinal qualities of tarthuth from local Muslim physicians. When the Crusaders lost Jerusalem to the Muslims, they moved to the island of Malta where *Cynomorium *was also native. The site where the "Maltese Mushroom" grew (Fungus Rock) was thereafter vigorously guarded and thieves were imprisoned or made galley slaves. The "treasure of drugs," as the Arabs called it, was used for a variety of purposes, including treating apoplexy, venereal disease, high blood pressure, vomiting, irregular menstrual periods and as a contraceptive and toothpaste.

Modern biomedical and phytochemical research on *Cynomorium coccineum *has demonstrated a variety of activities from plant extracts. Effects of *Cynomorium *extracts on mammalian reproductive cells modulation of pituitary gonadotrophins [37] as well as changes in testicular development [38] and epididymal sperm patterns [39] in rats. *Cynomorium songaricum*, known in Chinese medicine as "suo yang," has been shown to contain triterpenes with HIV protease inhibitory activity [40,41]. Interest in herbal medicines is growing at a rapid pace, and attention has been focused upon *Cynomorium *as evidenced by its availability via hundreds of distributors advertising (via the internet) herbal remedies for kidney and intestinal ailments as well as for impotence. Although it is not at present clear how many original distributors of the plant exist, and whether plant material being marketed as *Cynomorium *is actually this plant, what is clear is that it is not being cultivated, thus authentic herbal preparations must be obtained from wild populations. Very little information exists on the cultivation of *Cynomorium *[25] and certainly commercial suppliers are not practicing sustainable harvest by cultivating this obligate holoparasite. There is evidence that overexploitation of this plant has resulted in localized extinction [42]. For these reasons, we here raise concerns for the conservation of both species of *Cynomorium *and strongly voice the need to develop cultivation methodologies. Given the potential and actual biomedical applications of extracts from this plant, and conservation concerns given extensive harvesting from wild populations, information on its phylogenetic position within angiosperms is timely. Further molecular work will likely illuminate its closest relatives within Saxifragales. These taxa should then become the subject of phytochemical analyses to determine whether they also contain compounds of biomedical interest. Cultivation of photosynthetic plants would be more straightforward than the holoparasite, thus possibly relieving some of the pressure to harvest this more sensitive species from the wild.

## Conclusion

All previous classifications have allied *Cynomorium *and Balanophoraceae, likely owing to the holoparasitic habit and the presence of inflorescences with numerous tiny flowers. Molecular phylogenetic analyses using nuclear and mitochondrial gene sequences both indicate that these taxa are not closely related and that perceived similarities are a result of convergent evolution. *Cynomorium *is strongly supported as being a member of Saxifragales, however, its exact position within this order remains unresolved. Surprisingly, both nuclear and mitochondrial genes place Balanophoraceae with the sandalwood order, a relationship previously proposed but explained by some as a case of convergent evolution. Both species of wild *Cynomorium *are being harvested from wild populations for use in herbal medicines, and evidence exists for overexploitation. Given that methods to cultivate these holoparasites are not being employed, we here raise conservation concerns. If further molecular phylogenetic work identifies the nearest photosynthetic relatives of *Cynomorium*, these plants should be examined for phytochemical activity.

## Methods

### Taxon sampling and data collection

Voucher information for all newly sequenced taxa is given in Additional Files 1 and 2. DNA was extracted, PCR amplified, cloned, and sequenced following reported methods [3,43]. The nuclear and mitochondrial sequences were amplified using primers reported elsewhere [14,44,45]. Sequencing was conducted using an ABI Prism^® ^377 automated DNA sequencer (Applied Biosystems) according to the manufacturer's protocols. Two multiple sequence alignments were constructed. The purpose of the ***global ***data set was to test the position of *Cynomorium *and the three Balanophoraceae genera among angiosperm orders. This matrix included nuclear small-subunit (SSU) rDNA, chloroplast *rbcL *and *atpB*, and mitochondrial *matR *for 67 taxa with *Laurus *and *Cinnamomum *as outgroups. Balanophoraceae and Cynomorium lack the chloroplast genes *rbcL *and *atpB*, but they were included in the global data set to stabilize the overall tree topology as previously demonstrated [2,3]. Given the strong support for a relationship between *Cynomorium *and Saxifragales obtained from this global analysis, a ***Saxifragales ***data set was constructed to further resolve the position of the parasites within this clade. Here the genes used were nuclear SSU and LSU rDNA, chloroplast *rbcL*, *atpB*, and *matK *for 62 taxa with *Lea *and *Vitis *as outgroups. In both alignments, chloroplast genes were coded as missing for the holoparasites. Accession numbers for newly sequenced genes are: AY957440 – AY957454.

### Phylogenetic analyses

The ***global ***and ***Saxifragales ***data sets were analyzed using maximum parsimony (MP) in PAUP* 4.0b10 [46] and Bayesian inference (BI) methods in MrBayes 3.0b4 [47]. MP searches were performed using 100 random addition sequence replicates with tree-bisection-reconnection (TBR) branch-swapping, holding ten trees at each addition step, with all sites equally weighted. Fully partitioned Bayesian analyses were performed by partitioning each data set by gene and, for the protein-coding genes, by codon position. This resulted in a total of 10 partitions for the full data set and 11 partitions for the Saxifragales data set (Table 1). MP trees were constructed for each gene (following the protocol described above), and these trees were used in PAUP* to evaluate 24 nucleotide substitution models for each data partition. For example, models were evaluated for the "*rbcL *Pos1" partition (the first codon position of the *rbcL *data set) on the MP tree for the *rbcL *data set. MrModelTest 2.0 [48] was used to select an appropriate model from the PAUP* output via a second-order version of the Akaike Information Criterion (AIC*c*) that takes sample size into account, as recommended by Posada and Buckley [49]. AIC*c *values were computed using both the total number of characters and the number of variable characters per partition. For nearly all partitions, the Akaike weight for the chosen model was much higher than the Akaike weight of the next best model, so model-averaged analyses were not performed. The best-fitting models and their Akaike weights for each data partition are listed in Supplementary Data. Partitioned Bayesian analyses were performed with all model parameters unlinked (i.e., model parameters for each data partition were estimated separately from each partition), and with topology and branch lengths linked. Two separate analyses (with different MCMC seeds, random starting trees and default uniform priors for all parameters) were run for each data set for 15 million generations, with trees sampled every 500 generations. Trees recovered during the first 2.5 million generations (the first 5000 trees) in both runs were discarded as burn-in, leaving a total of 25,000 trees which were used to construct majority-rule consensus trees.

**Table 1:** Summary of output from MrModelTest for each data partition in the *global *and *Saxifragales *data sets §

**Data Set**	**Data Partition**	**Chosen Model (AICc)**	**Chosen Model (hLRT)**	**Characters (total/variable)**	**Akaike Weight of Chosen Model**
Both *global *and *Saxifragales*	*atpB *Pos3	GTR+I+Γ	GTR+I+Γ/GTR+Γ	490/416	0.9997 (all)
	*rbcL *Pos1	GTR+I+Γ	GTR+I+Γ	467/130	1.0000 (all)
	*rbcL *Pos2	SYM+I+Γ	SYM+I+Γ/JC+I+Γ	467/84	0.7397 (all)
	*rbcL *Pos3	GTR+I+Γ	GTR+I+Γ	467/391	0.9911 (all)
	nu SSU	GTR+I+Γ	SYM+I+Γ/GTR+I+Γ	1750/428	0.8264 (all)

Just *global*	*atpB *Pos1*	GTR+I+Γ	GTR+I+Γ	490/139	1.0000 (all)
	*atpB *Pos2*	GTR+I+Γ	GTR+I+Γ	490/68	0.9872 (all)
	*matR *Pos1	GTR+Γ	GTR+Γ	743/298	0.6598 (all)
	*matR *Pos2	GTR+Γ	GTR+Γ/HKY+Γ	743/285	0.5628 (all)
	*matR *Pos3	GTR+Γ	SYM+Γ/GTR+Γ	742/376	0.6739 (all)

Just *Saxifragales*	*atpB *Pos1*	GTR+G	GTR+G/GTR+I	480/85	0.4127 (all)
	*atpB *Pos2*	GTR+I+Γ (all)/ HKY+I+Γ (variable)	HKY+I/HKY+I+Γ/ GTR+I+Γ	480/45	0.3988 (variable)
	*matK *Pos1	GTR+I+Γ	GTR+Γ	528/297	0.5389 (all)
	*matK *Pos2	GTR+I+Γ	GTR+I+Γ/GTR+Γ	528/250	0.9208 (all)
1.	*matK *Pos3	GTR+Γ	GTR+Γ	528/369	0.7387 (all)
	nu LSU	GTR+I+Γ	GTR+I+Γ	3391/808	1.0000 (all)

§Models selected by the hLRT can vary depending on the model parameter addition hierarchy used; models used for analysis were those chosen by the AICc and at least one version of the hLRT [for *matK *pos 1, AICc was used]. The listed numbers of variable characters in the "both *global *and *Saxifragales*" portion of the table refer to the *global *data set. Akaike weights of the chosen model were computer for the total number of characters (all) and the number of variable characters (variable). * Both the *global *and *Saxifragales *data sets included *atpB *codon positions 1 and 2, but the models chosen for these data partitions differed between the two data sets (simpler models were chosen for these partitions with the *Saxifragales *data set, which comprises more closely related taxa than does the *global *data set).

## List of abbreviations

AIC*c *– Akaike Information criterion

*atpB *– ATP synthase beta subunit

BI – Bayesian inference

BS – bootstrap

GTR – general time reversible model (a model of DNA sequence evolution)

HIV – human immunodeficiency virus

I + Γ – invariant sites plus gamma distribution

*matK *– chloroplast maturase K

*matR *– mitochondrial maturase R

MCMC – Markov Chain Monte Carlo (a simulation method used to approximate the posterior probabilities of trees)

ML – maximum likelihood

MP – maximum parsimony

*rbcL *– ribulose bisphosphate carboxylase/oxygenase, large subunit

nu SSU – nuclear small-subunit ribosomal DNA

nu LSU – nuclear large-subunit ribosomal DNA

TBR – tree bisection-reconnection branch swapping

## Authors' contributions

DLN coordinated all aspects of the study, obtained directly or through colleagues all genomic DNAs used herein, conducted sequence aligments, and drafted the manuscript. JPD obtained all of the newly generated sequences. FEA performed all phylogenetic analyses. All of the authors read and approved the final manuscript.

## Supplementary Material

Additional File 1 **Taxon sampling – global data set. **MS Excel file giving taxon sampling by gene and GenBank accession numbers for the ***global ***data set. Click here for file

Additional File 2 **Taxon sampling – Saxifragales data set. **MS Excel file giving taxon sampling by gene and GenBank accession numbers for the ***Saxifragales ***data set. Click here for file

Additional File 3 **BI tree from global data set. **Bayesian inference majority rule consensus tree of 50,000 trees derived from the *global *data set composed of nuclear SSU rDNA, chloroplast *rbcL*, *atpB*, and mitochondrial *matR*. Trees were generated in two separate BI analyses, each run for 15 million generations with trees from the first 2.5 million generations removed as burn-in. Click here for file

Additional File 4 **BI tree from the nuclear SSU rDNA partition. **Bayesian inference majority rule consensus tree of 50,000 trees derived from the nuclear SSU rDNA partition. Trees were generated in two separate BI analyses, each run for 15 million generations with trees from the first 2.5 million generations removed as burn-in. Click here for file

Additional File 5 **ML tree from the nuclear SSU rDNA partition. **Phylogram from maximum likelihood (ML) analysis of the nuclear SSU rDNA partition (GTR+I+Γ model. The tree was generated using a successive approximations approach in which a MP tree was generated and used as a starting tree for additional branch swapping under ML. Click here for file

Additional File 6 **BI tree from the mitochondrial matR partition. **Bayesian inference majority rule consensus tree of 50,000 trees derived from the mitochondrial *matR *partition. Trees were generated in two separate BI analyses, each run for 15 million generations with trees from the first 2.5 million generations removed as burn-in. Click here for file

Additional File 7 **BI tree from the Saxifragales data set. **Bayesian inference majority rule consensus tree of 50,000 trees derived from the *Saxifragales *data set. Trees were generated in two separate BI analyses, each run for 15 million generations with trees from the first 2.5 million generations removed as burn-in. Click here for file

Additional File 8 **MP phylogram from the global data set. **Data set of combined nuclear SSU rDNA, chloroplast *rbcL *and *atpB *and mitochondrial *matR*. Tree one of four is shown, with branch lengths drawn proportional to the number of changes. Click here for file

Additional File 9 **BI majority rule phylogram from the global data set. **Bayesian inference majority rule consensus phylogram from the *global *data set (nuclear SSU rDNA, chloroplast *rbcL *and *atpB *and mitochondrial *matR*). Branch lengths are means of the branch length posterior probability distribution across all post burn-in trees from one of the two MrBayes runs (branch lengths resulting from the other run are nearly identical). Click here for file

## References

[B1] APG (2003). An update of the Angiosperm Phylogeny Group classification for the orders and families of flowering plants: APG II. Botanical Journal of the Linnean Society London.

[B2] Nickrent DL, Blarer A, Qiu YL, Soltis DE, Soltis PS, Zanis M (2002). Molecular data place Hydnoraceae with Aristolochiaceae. Amer J Bot.

[B3] Nickrent DL, Blarer A, Qiu YL, Vidal-Russell R, Anderson FE (2004). Phylogenetic inference in Rafflesiales: the influence of rate heterogeneity and horizontal gene transfer. BMC Evolutionary Biology.

[B4] Wendel JF, Doyle JJ, Soltis DE, Soltis PS and Doyle JJ (1998). Phylogenetic incongruence: window into genome history and molecular evolution. Molecular Systematics of Plants II DNA Sequencing.

[B5] Davis CC, Wurdack KJ (2004). Host-to-parasite gene transfer in flowering plants: phylogenetic evidence from Malpighiales. Science.

[B6] Mower JP, Stefanovic S, Young GJ, Palmer JD (2004). Gene transfer from parasitic to host plants. Nature (London).

[B7] Hooker JD (1856). On the structure and affinities of Balanophoraceae. Trans Linn Soc London.

[B8] Cronquist A (1981). An integrated system of classification of flowering plants.

[B9] Takhtajan A (1997). Diversity and classification of flowering plants.

[B10] Kuijt J (1969). The Biology of Parasitic Flowering Plants.

[B11] Pazy B, Plitmann U, Cohen O (1996). Bimodal karyotype in Cynomorium coccineum L. and its systematic implications. Botanical Journal of the Linnean Society London.

[B12] Teryokhin ES, Yakovlev MS, Nikitcheva ZI (1975). Development of microsporangium, pollen grains, ovule and embryo sac in Cynomorium songaricum Rupr. (Cynomoriaceae). Botnicheskii Zhurnal.

[B13] Nickrent DL, López-Sáez JA, Catalán P and Sáez L (2002). Orígenes filogenéticos de las plantas parásitas. Plantas Parásitas de la Península Ibérica e Islas Baleares.

[B14] Nickrent DL, Starr EM (1994). High rates of nucleotide substitution in nuclear small-subunit (18S) rDNA from holoparasitic flowering plants. Journal of Molecular Evolution.

[B15] Fishbein M, Soltis DE (2004). Further resolution of the rapid radiation of Saxifragales (angiosperms, eudicots) supported by mixed-model Bayesian analysis. Systematic Botany.

[B16] Fishbein M, Hibsch-Jetter C, Soltis DE, Hufford L (2001). Phylogeny of Saxifragales (angiosperms, eudicots): Analysis of a rapid, ancient radiation. Systematic Biology.

[B17] Soltis DE, Senters AE, Zanis MJ, Kim S, Thompson JD, Soltis PS, Ronse De Craene LP, Endress PK, Farris JS (2003). Gunnerales are sister to other core eudicots: implications for the evolution of pentamery. Amer J Bot.

[B18] Hilu KW, Borsch T, Muller K, Soltis DE, Soltis PS, Savolainen V, Chase MW, Powell MP, Alice LA, Evans R, Sauquet H, Neinhuis C, Slotta TAB, Rohwer JG, Campbell CS, Chatrou LW (2003). Angiosperm phylogeny based on matK sequence information. American Journal of Botany.

[B19] Juel O (1910). Cynomorium und Hippuris. Svensk Bot Tidskr.

[B20] Soltis DE, Soltis PS, Chase MW, Mort ME, Albach DC, Zanis M, Savolainen V, Hahn WH, Hoot SB, Fay MF, Axtell M, Swensen SM, Prance LM, Kress WJ, Nixon KC, Farris JS (2000). Angiosperm phylogeny inferred from 18S rDNA, rbcL, and atpB sequences. Botanical Journal of the Linnean Society London.

[B21] Kim S, Soltis DE, Soltis PS, Zanis MJ, Suh Y (2004). Phylogenetic relationships among early-diverging eudicots based on four genes: were the eudicots ancestrally woody?. Molecular Phylogenetics and Evolution.

[B22] Judd WS, Olmstead RG (2004). A survey of tricolpate (eudicot) phylogenetic relationships. Amer J Bot.

[B23] Anderson FE, Swofford DL (2004). Should we be worried about long-branch attraction in real data sets? Investigations using metazoan 18S rDNA. Mol Phylo Evol.

[B24] Harborne JB, Saito N, Detoni CH (1994). Anthocyanins of Cephaelis, Cynomorium, Euterpe, Lavatera and Pinanga. Biochemical Systematics and Ecology.

[B25] Weddell HA (1860). Memoire sur le Cynomorium coccineum, parasite de l'ordre des Balanophorées. Archives du Muséum National d'Histoire Naturelle.

[B26] Engler A, Engler A and Prantl K (1894). Balanophoraceae. Die Naturlichen Pflanzenfamilien, Nachtr.

[B27] Fagerlind F (1948). Beiträge zür Kenntnis der Gynäceummorphologie und Phylogenie der Santalales-Familien. Svensk Botanisk Tidskrift.

[B28] Takhtajan AL (1980). Outline of the classification of flowering plants (Magnoliophyta). Botanical Review.

[B29] Thorne RF (1992). An updated phylogenetic classification of the flowering plants. Aliso.

[B30] Kuijt J (1968). Mutual affinities of Santalalean families. Brittonia.

[B31] Nickrent DL, Malécot V, Fer A, Thalouarn P, Joel DM, Musselman LJ, Parker C and Verkleij JAC (2001). A molecular phylogeny of Santalales. Proceedings of the 7th International Parasitic Weed Symposium.

[B32] Weber HC (1986). Granulahaltige Xylem-Leitbahnen und andere den Santalales ähnliche anatomische Strukturen in den haustorialen Knollen von Mystropetalon thomii Harv. (Balanophoraceae). Flora.

[B33] Bhandari NN, Vohra SCA, Calder M and Bernhardt P (1983). Embryology and affinities of Viscaceae. The Biology of Mistletoes.

[B34] Hull RJ, Leonard OA (1964). Physiological aspects of parasitism in mistletoes (Arceuthobium and Phoradendron). 1.   The carbohydrate nutrition of mistletoe. Pl Phys.

[B35] Hull RJ, Leonard OA (1964). Physiological aspects of parasitism in mistletoes (Arceuthobium and Phoradendron). 2. The photosynthetic capacity of mistletoes. Pl Phys.

[B36] Lebling RW (2003). The treasure of tarthuth. Saudi Aramco World.

[B37] Al-Qarawi AA, Abdel-Rahman HA, El-Badry AA, Harraz F, Razig NA, Abdel-Magied EM (2000). The effect of extracts of Cynomorium coccineum and Withania somnifera on gonadotrophins and ovarian follicles of immature Wistar rats. Phytother Res.

[B38] Abd el-Magied EM, Abd el-Rahman HA, Harraz FM (2001). The effect of aqueous extracts of Cynomorium coccineum and Withania somnifera on testicular development in immature Wistar rats. J Ethnopharmacol.

[B39] Abd el-Rahman HA, El-Badry AA, Mahmoud OM, Harraz FA (1999). The effect of the aqueous extract of Cynomorium coccineum on the epididymal sperm pattern of the rat. Phytother Res.

[B40] Ma CM, Nakamura N, Miyashiro H, Hattori M, Shimotohno K (1999). Inhibitory effects of constituents from Cynomorium songaricum and related triterpene derivatives on HIV-1 protease. Chem Pharm Bull.

[B41] Nakamura N (2004). Inhibitory effects of some traditional medicines on proliferation of HIV-1 and its protease. Yakugaku Zasshi.

[B42] Xie Y, Wang S (2001). Chapter 11: People and biodiversity.

[B43] Nickrent DL (1994). From field to film: rapid sequencing methods for field collected plant species. BioTechniques.

[B44] Qiu Y, Lee Y, Bernasconi-Quadroni F, Soltis DE, Soltis PS, Zanis M, Zimmer EA, Chen Z, Savolainen V, Chase MW (1999). The earliest angiosperms: evidence from mitochondrial, plastid and nuclear genomes. Nature.

[B45] Kuzoff RK, Sweere JA, Soltis DE, Soltis PS, Zimmer EA (1998). The phylogenetic potential of entire 26S rDNA sequences in plants. Mol Biol Evol.

[B46] Swofford DL (2002). PAUP*: phylogenetic analysis using parsimony (* and other methods).

[B47] Ronquist F, Huelsenbeck JP (2003). MrBayes 3: Bayesian phylogenetic inference under mixed models. Bioinformatics.

[B48] Nylander JA (2004). MrModelTest.

[B49] Posada D, Buckley TR (2004). Model selection and model averaging in phylogenetics: advantages of Akaike information criterion and Bayesian approaches over likelihood ratio tests. Systematic Biology.

