# Supplementary figures and images for: Discovery of the photosynthetic relatives of the "Maltese mushroom" Cynomorium
Source: BMC Evol Biol. 2005 Jun 21;5:38. doi: 10.1186/1471-2148-5-38 (PMC1182362; doi:10.1186/1471-2148-5-38)

Additional File 3

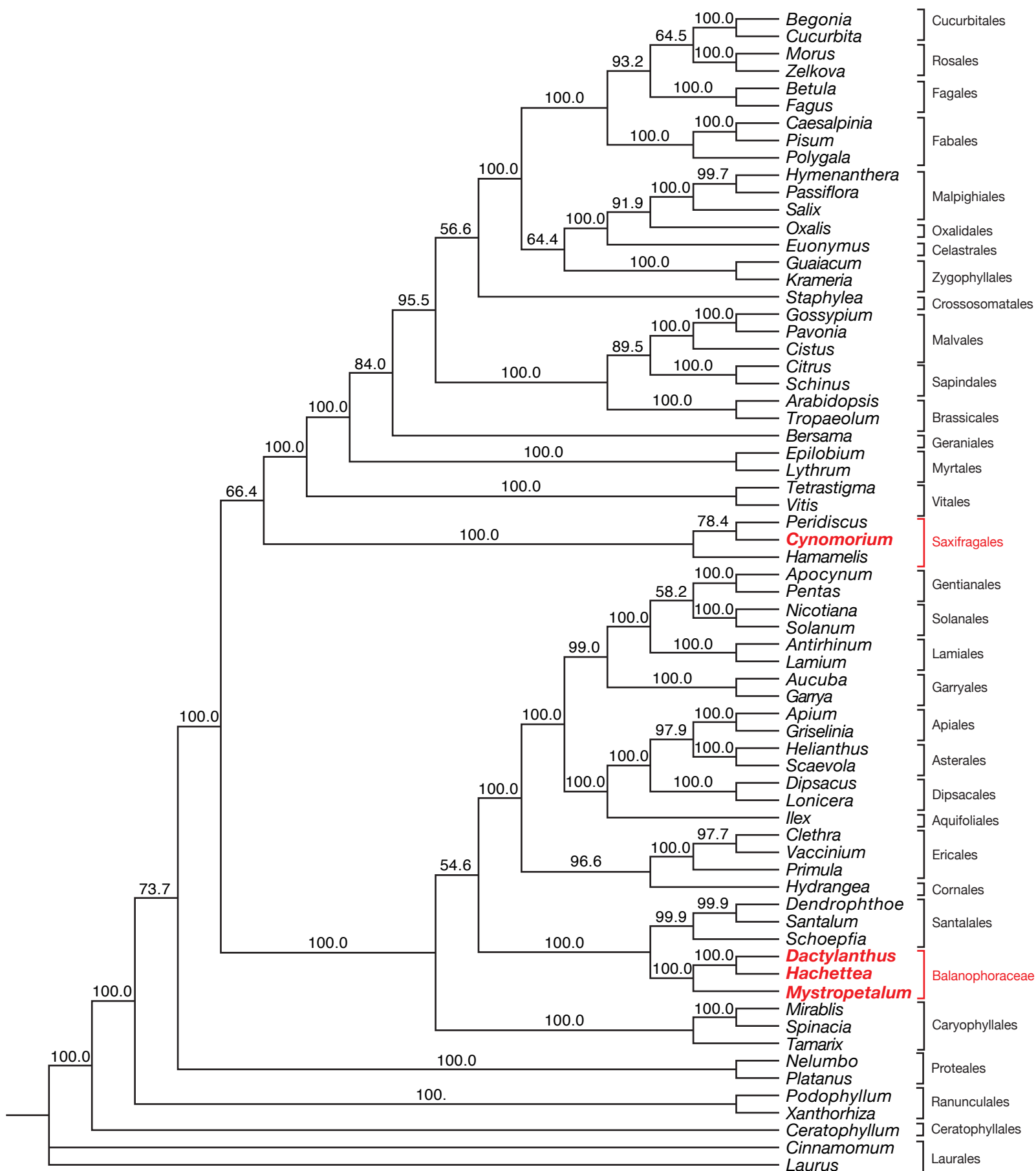

Supplement: Additional File 3 — BI tree from global data set. Bayesian inference majority rule consensus tree of 50,000 trees derived from the global data set composed of nuclear SSU rDNA, chloroplast rbcL, atpB, and mitochondrial matR. Trees were generated in two separate BI analyses, each run for 15 million generations with trees from the first 2.5 million generations removed as burn-in. [file 1471-2148-5-38-S3.PDF]

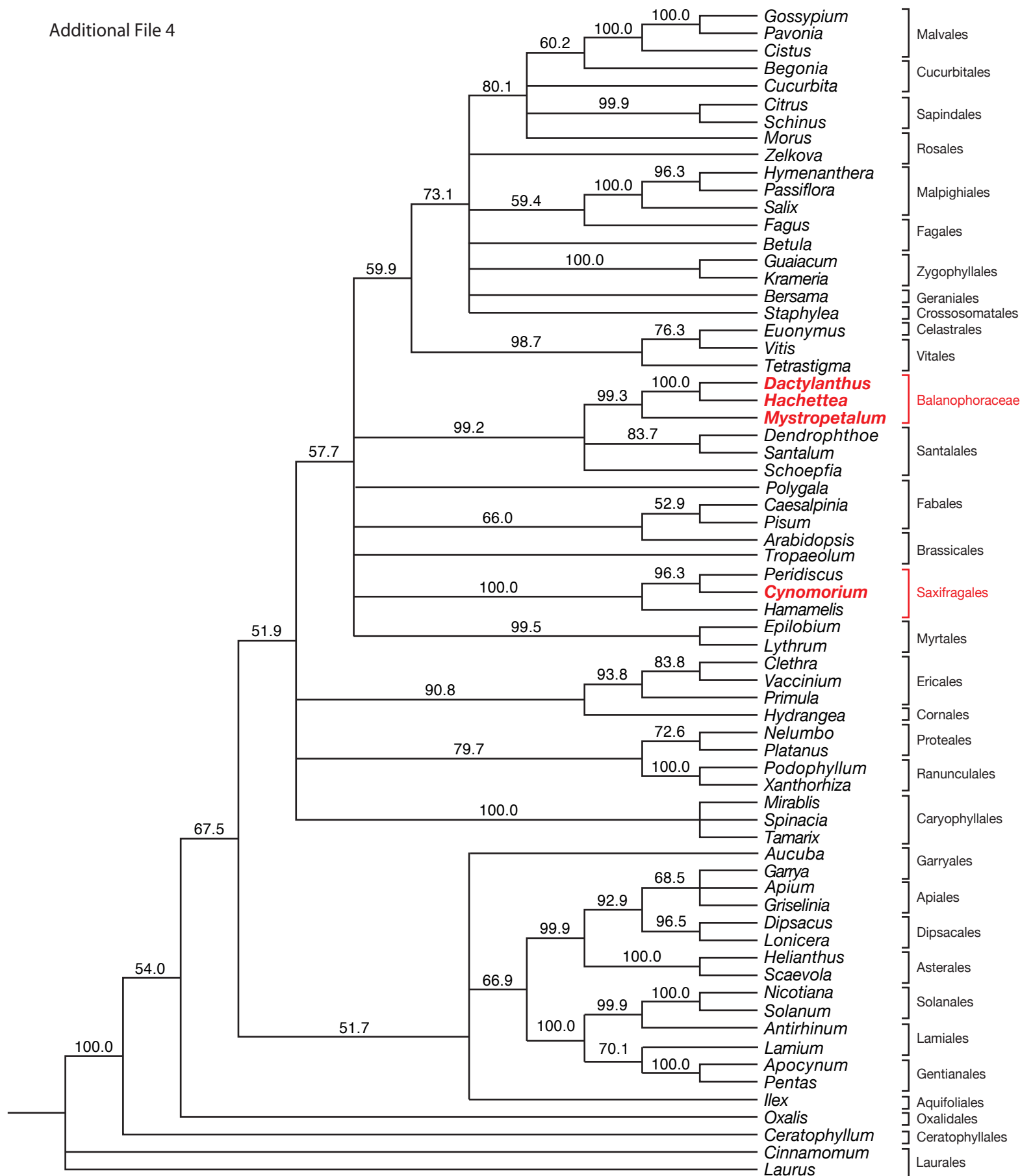

Supplement: Additional File 4 — BI tree from the nuclear SSU rDNA partition. Bayesian inference majority rule consensus tree of 50,000 trees derived from the nuclear SSU rDNA partition. Trees were generated in two separate BI analyses, each run for 15 million generations with trees from the first 2.5 million generations removed as burn-in. [file 1471-2148-5-38-S4.PDF]

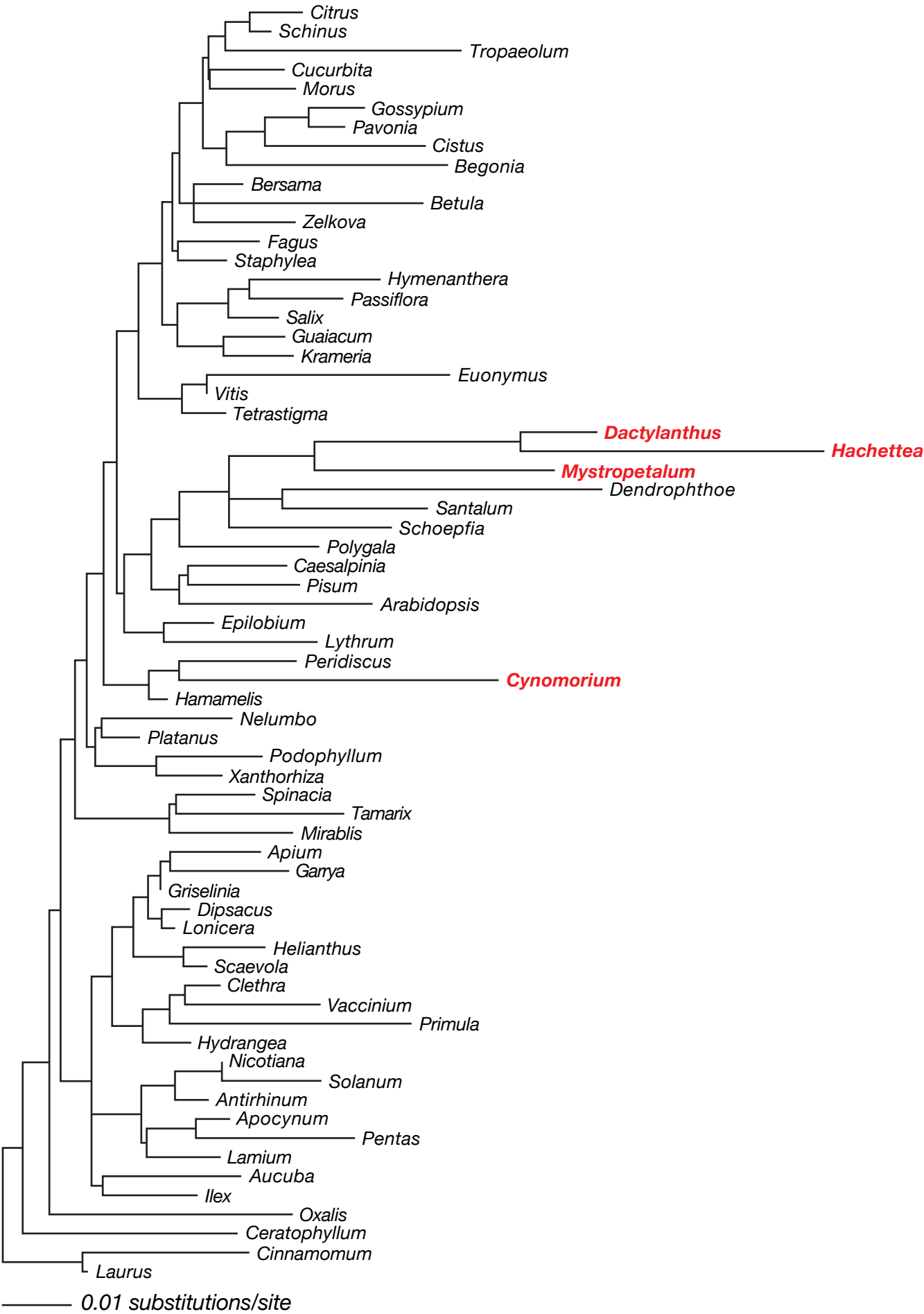

Supplement: Additional File 5 — ML tree from the nuclear SSU rDNA partition. Phylogram from maximum likelihood (ML) analysis of the nuclear SSU rDNA partition (GTR+I+Γ model. The tree was generated using a successive approximations approach in which a MP tree was generated and used as a starting tree for additional branch swapping under ML. [file 1471-2148-5-38-S5.PDF]

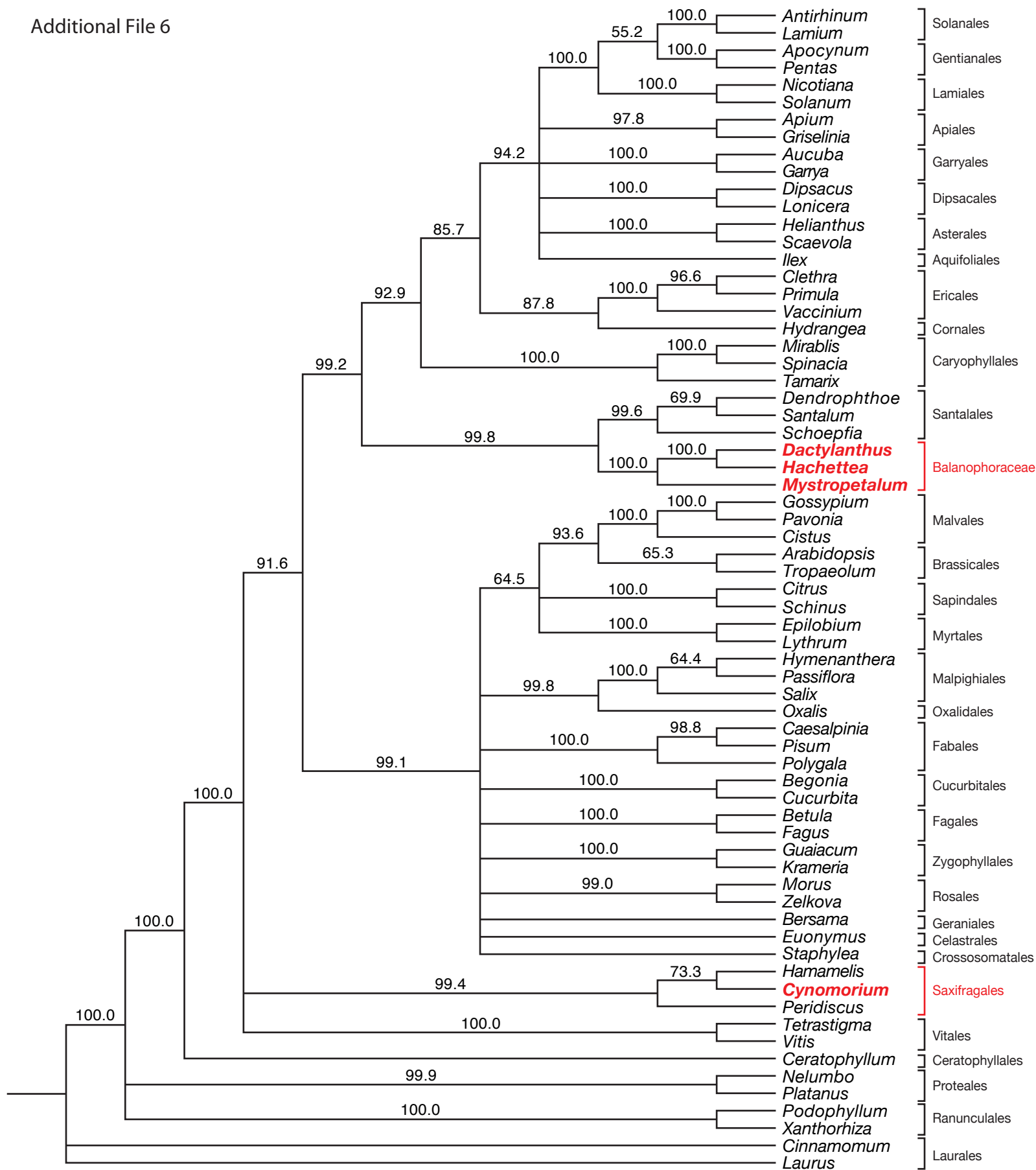

Supplement: Additional File 6 — BI tree from the mitochondrial matR partition. Bayesian inference majority rule consensus tree of 50,000 trees derived from the mitochondrial matR partition. Trees were generated in two separate BI analyses, each run for 15 million generations with trees from the first 2.5 million generations removed as burn-in. [file 1471-2148-5-38-S6.PDF]

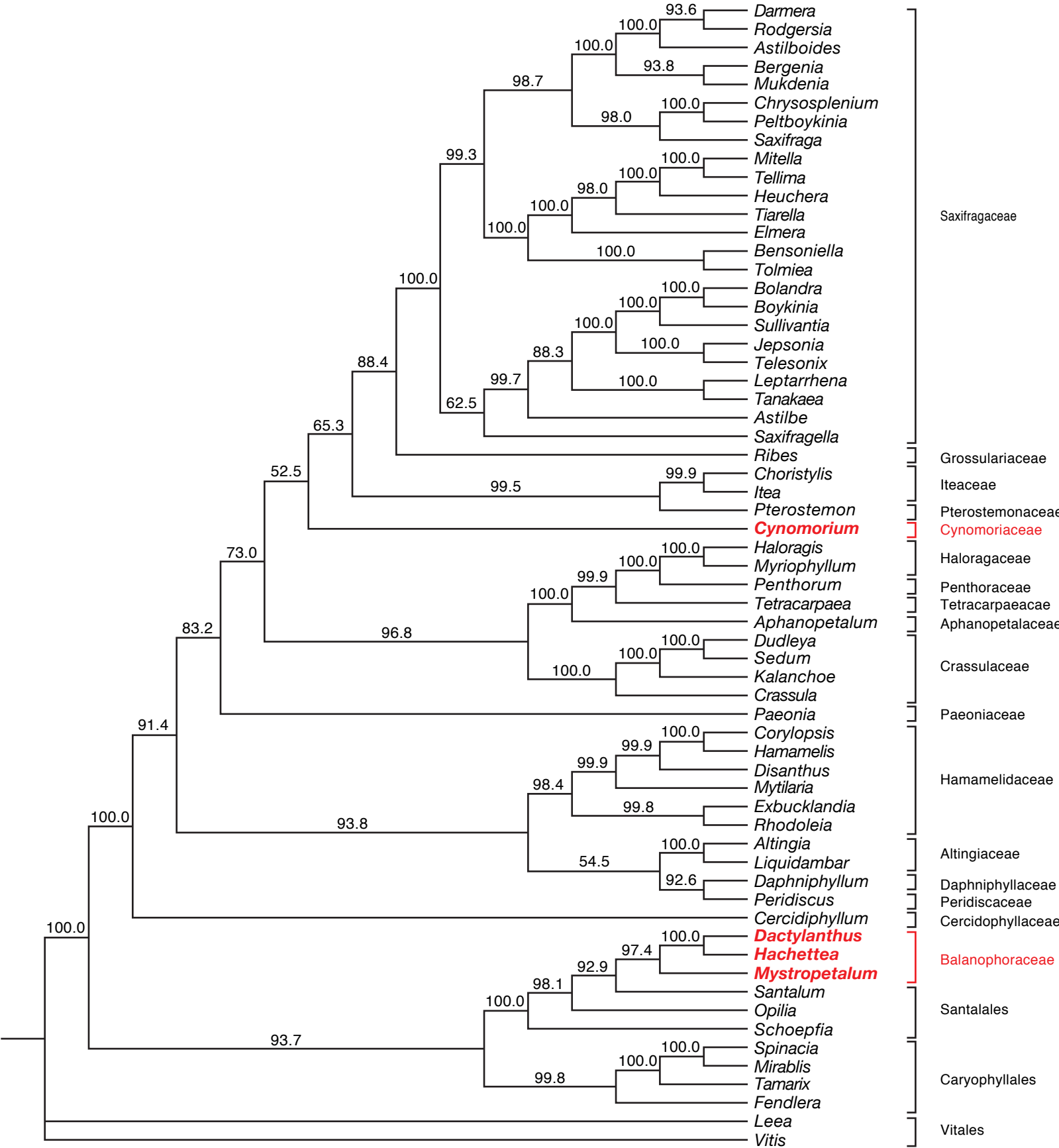

Supplement: Additional File 7 — BI tree from the Saxifragales data set. Bayesian inference majority rule consensus tree of 50,000 trees derived from the Saxifragales data set. Trees were generated in two separate BI analyses, each run for 15 million generations with trees from the first 2.5 million generations removed as burn-in. [file 1471-2148-5-38-S7.PDF]

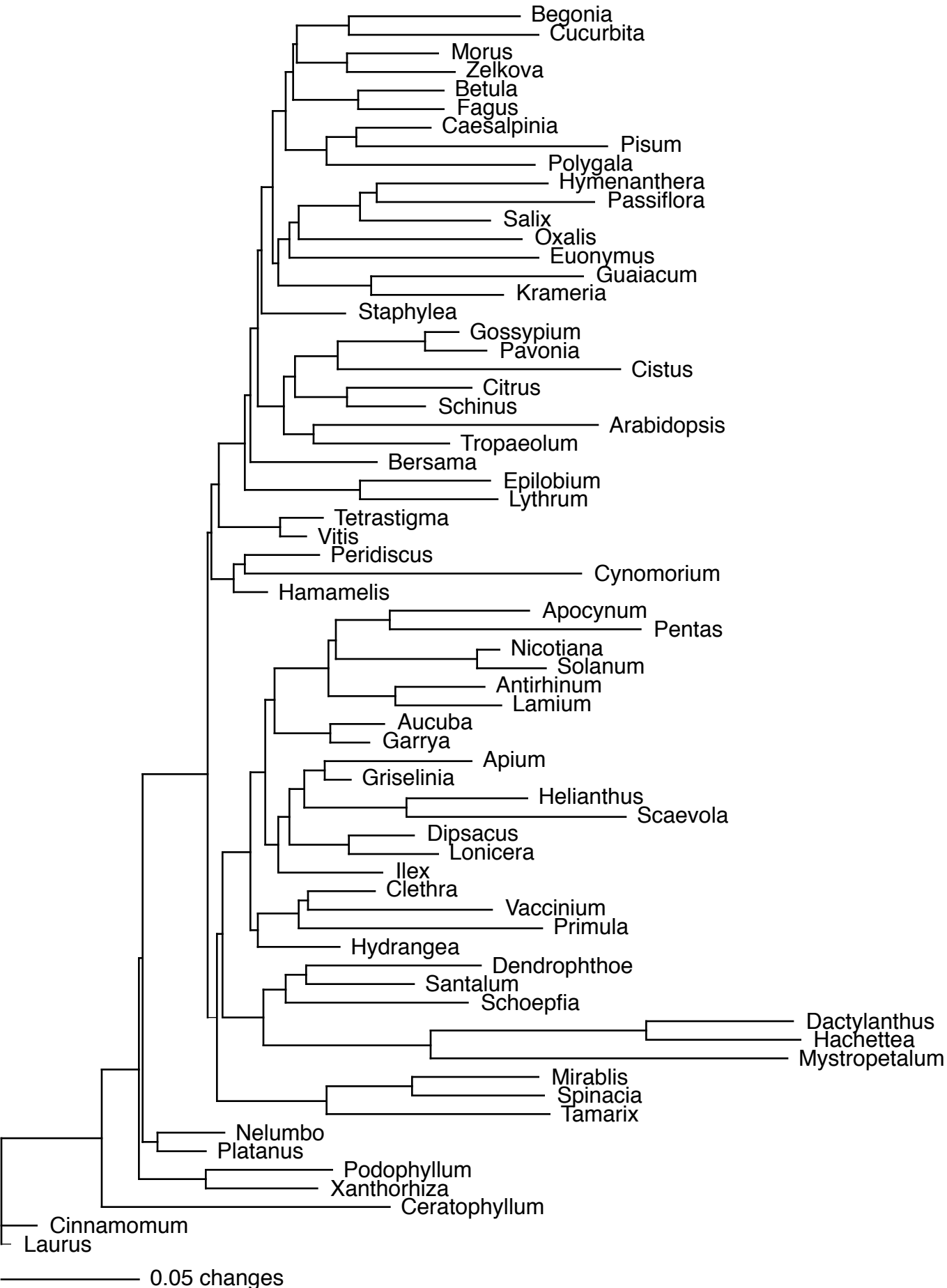

Supplement: Additional File 8 — MP phylogram from the global data set. Data set of combined nuclear SSU rDNA, chloroplast rbcL and atpB and mitochondrial matR. Tree one of four is shown, with branch lengths drawn proportional to the number of changes. [file 1471-2148-5-38-S8.PDF]

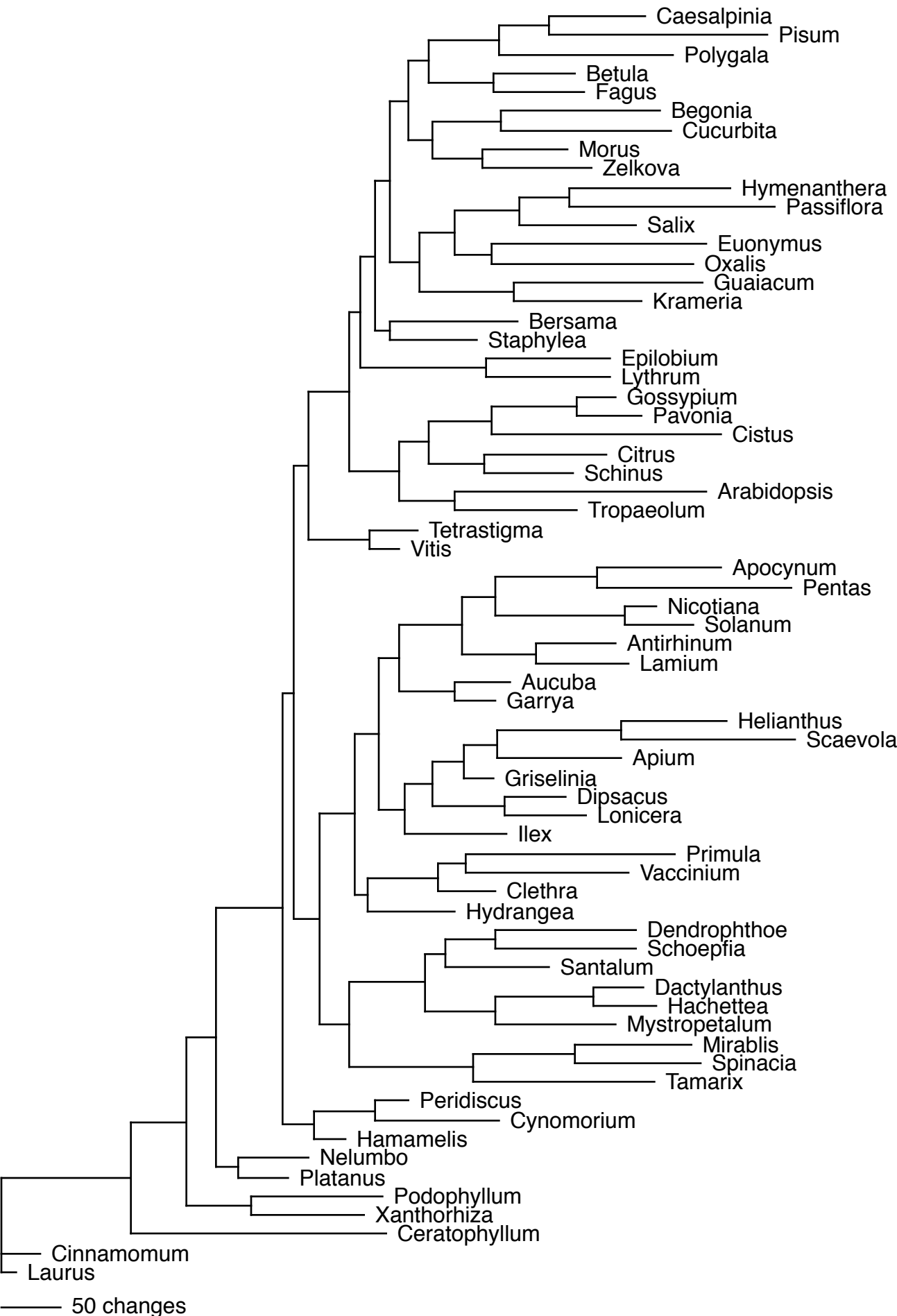

Supplement: Additional File 9 — BI majority rule phylogram from the global data set. Bayesian inference majority rule consensus phylogram from the global data set (nuclear SSU rDNA, chloroplast rbcL and atpB and mitochondrial matR). Branch lengths are means of the branch length posterior probability distribution across all post burn-in trees from one of the two MrBayes runs (branch lengths resulting from the other run are nearly identical). [file 1471-2148-5-38-S9.PDF]
